# Deferasirox Nanosuspension Loaded Dissolving Microneedles for Intradermal Delivery

**DOI:** 10.3390/pharmaceutics14122817

**Published:** 2022-12-15

**Authors:** Hafsa Shahid Faizi, Lalitkumar K. Vora, Muhammad Iqbal Nasiri, Yu Wu, Deepakkumar Mishra, Qonita Kurnia Anjani, Alejandro J. Paredes, Raghu Raj Singh Thakur, Muhammad Usman Minhas, Ryan F. Donnelly

**Affiliations:** 1 School of Pharmacy, Queen’s University Belfast, Medical Biology Centre, 97 Lisburn Road, Belfast BT9 7BL, UK; 2 Department of Pharmaceutics, Hamdard Institute of Pharmaceutical Sciences, Hamdard University, Islamabad 45550, Pakistan; 3 College of Pharmacy, University of Sargodha, Sargodha 40100, Pakistan

**Keywords:** nanocrystals, nanosuspension, dissolving microneedles, deferasirox, intradermal delivery

## Abstract

Microneedles are minimally invasive systems that can deliver drugs intradermally without pain and bleeding and can advantageously replace the hypodermal needles and oral routes of delivery. Deferasirox (DFS) is an iron chelator employed in several ailments where iron overload plays an important role in disease manifestation. In this study, DFS was formulated into a nanosuspension (NSs) through wet media milling employing PVA as a stabilizer and successfully loaded in polymeric dissolving microneedles (DMNs). The release studies for DFS-NS clearly showed a threefold increased dissolution rate compared to pure DFS. The mechanical characterization of DFS-NS-DMNs revealed that the system was sufficiently strong for efficacious skin penetration. Optical coherence tomography images confirmed an insertion of up to 378 µm into full-thickness porcine skin layers. The skin deposition studies showed 60% drug deposition from NS-DMN, which was much higher than from the DFS-NS transdermal patch (DFS-NS-TP) (without needles) or pure DFS-DMNs. Moreover, DFS-NS without DMNs did not deposit well inside the skin, indicating that DMNs played an important role in effectively delivering drugs inside the skin. Therefore, it is evident from the findings that loading DFS-NS into novel DMN devices can effectively deliver DFS transdermally.

## 1. Introduction

In recent times, the inadequacy of drugs’ cross through the skin barrier, the stratum corneum (SC), remains a major limitation of transdermal delivery [1,2]. This problem has been addressed by introducing micron-scale needles that can enhance skin permeability, thus increasing effective transdermal delivery [3,4]. Microneedles (MNs) that employ a combination of hypodermic needles and transdermal patches are painless and negligibly invasive devices that can bypass the SC [5,6]. As the *SC* contains no nociceptors and MNs do not invade deeper where nerve endings are present, they are capable of carrying drugs to the permeable regions of skin without provoking nerves responsible for pain [7,8]. MNs can also obviate the first pass effect, which is a classic drawback of oral drug delivery, by effectively delivering drugs via the intradermal route [9,10].

DFS is an iron chelator employed in iron toxicity for various diseases [11]. It is highly effective in treating iron overload in thalassemic patients caused by blood transfusions [12,13]. DFS has also been investigated for reducing oxidative stress and inflammation in patients where iron is responsible for the development of inflammation and tissue damage through the production [14] of reactive oxygen species (ROS). It forms a stable complex with Fe(III) ions with 2:1 binding to eliminate iron. DFS, due to its iron-chelating properties, is proven to be safe and effective in the treatment of a skin condition called porphyria cutanea tarda [15]. Other studies found that DFS presented antitumor activity for treating solid tumors [16,17]. DFS is a Class II compound, according to the biopharmaceutics classification system (BCS), exhibiting low solubility and high permeability [12,18]. Current oral drug delivery can result in the short duration of action, resulting in a higher dosing frequency with lower patient compliance. In addition to the fact that first pass effect decreases the bioavailability of drug, it can also be unsuitable for patients who are unconscious and/or vomiting. Therefore, an alternative drug delivery route is highly desirable. To avoid the stated drawbacks for oral route, intradermal delivery improved therapy due to the maintenance of plasma levels up to the end of the dosing interval compared to a decline in plasma levels with oral delivery, making it a major advantage of the former. However, impermeable skin barrier could not allow the delivery of the hydrophobic drug as its absorption in the viable layers of the skin is negligible. One drug delivery strategy that could be considered to improve the delivery of this hydrophobic DFS into the skin are MNs, which circumvent the protective barrier of the *SC* by physically piercing this outermost layer of the skin [19,20,21].

However, unlike hydrophilic drugs, the formation of nanosuspensions (NSs) of hydrophobic drugs is necessary for inclusion into polymeric MNs for uniform distribution inside MNs [19,22,23,24]. NSs refer to a nanosized (1–1000 nm) liquid dispersion of drug particles coated by a stabilizer layer, for example, a surfactant or polymer [25,26,27]. Additionally, the advantages of increased surface area, greater dissolution rates, better absorption and, hence, higher bioavailability, make nanosuspension a desirable and widely employed technique for hydrophobic drugs [28,29,30]. Moreover, NS can yield high drug loading because of the smaller amount of surfactants needed to stabilize nanosized drug molecules, unlike polymeric nanoparticles, where the use of larger amounts of polymeric excipients is essential to encapsulate the drug molecules [31,32].

In this study, two concepts of drug nanosizing and microneedle-based intradermal drug delivery were combined to enhance the efficient delivery of the poorly water-soluble drug DFS. The goal of this work was the incorporation of DFS NSs into polymeric MNs for effective intradermal delivery as opposed to oral delivery, as it presents numerous drawbacks. First, DFS NSs were prepared using wet media milling. Afterward, the DFS-NS were subsequently loaded into the DMNs. The newly formed MNs, after effective loading of drug NSs, were assessed for particle size, polydispersity index (PDI), mechanical strength, skin insertion, drug content, and ex vivo skin deposition of the drug.

## 2. Materials and Methods

### 2.1. Materials

Deferasirox (DFS) was purchased from Cangzhou Enke Pharma-tech Co., Ltd., Guangzhou, China. Poly(vinyl alcohol) (PVA) (9000–10,000 mol wt) was purchased from Sigma Aldrich (Poole, Dorset, UK). Poly(vinyl pyrrolidone) (PVP) with a molecular weight of 58,000 Da (K-29/32) and a PVP molecular weight of 111. A total of 143 Da (K-90) were purchased from Ashland Industries (Wilmington, DE, USA). The purified water utilized in all experiments was obtained from ELGA^®^ DV 25, Purelab Option, water purification System (ELGA-Q, USA). All other chemicals used were of analytical reagent grade.

### 2.2. Preparation of Drug-Loaded Nanosuspension

**Figure 1:**
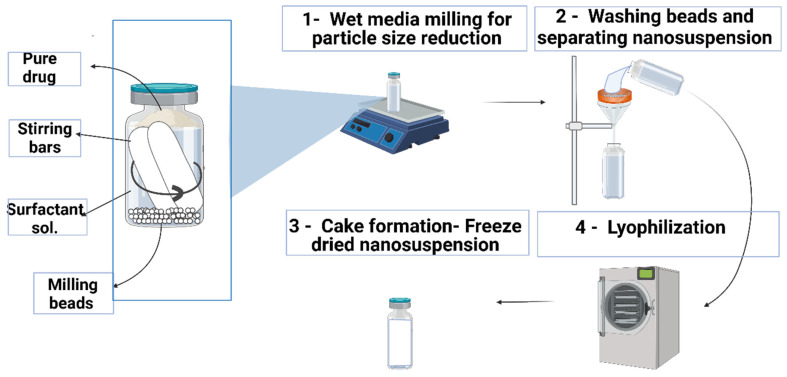
Schematic representation of the fabrication of the DFS-NS.

DFS NSs were prepared with the wet media milling method, as presented in Figure 1, using beads (ceramic beads, 0.2 mm diameter) with slight modifications, as reported previously [33]. This preparation method was chosen because of its easy and solvent-free operation and high drug loading [1]. PVA was selected as a stabilizer for the NS. One hundred milligrams of DFS was accurately weighed in a 7 mL volume glass vial, followed by the addition of 6 mL of 1% *w*/*w* PVA solution. Approximately 2 mL of beads was added to this mixture, and two magnetic stirring bars of dimensions (25 mm × 8 mm) were placed in the vial. The vial containing drug, stabilizer, ceramic beads (particle size of 0.1 mm) and magnetic stirrers was placed on a magnetic stirring plate running at a speed of 1000 rpm and 1500 rpm for 6–12 h. The resulting NS were retrieved after 24 h of milling, kept at −80 °C for 6–8 h and freeze-dried at −40 °C for 26 h to remove all water content. The effect of an increase in milling speed and milling time on particle size was also recorded.

### 2.3. Particle Size and Surface Charge Analysis

The hydrodynamic size, zeta potential, and polydispersity index (PDI) of freshly prepared and lyophilized DFS-NS were determined using a NanoBrook Omni (Brookhaven Instrument, Holtsville, NY, USA). PDI shows uniformity in the size distribution of particles within a sample, and zeta potential gives us information on the stability of the given formulation. Freshly prepared DFS-NS samples were taken at 6, 12, 24 and 48 h to monitor particle size reduction as a function of milling time. Briefly, samples were diluted suitably in water prior to measurement. An electric field was applied across the DFS-NS solutions using the technique of phase analysis light scattering (PALS) procedure for measuring zeta potential. Particle size and PDI were analyzed by employing the dynamic light scattering (DLS) method. Folded capillary cells were used for holding samples, and the temperature was maintained at 25 ± 2 °C for each measurement. All experimental runs were performed in triplicate to obtain mean data.

### 2.4. Particle Size Analysis for Pure Drug

For determination of DFS particle size, a Mastersizer^®^ 3000 equipped with a Hydro^®^ cell (Malvern Panalytical Ltd., Worcestershire, UK) was used employing laser diffraction phenomenon, as previously described [34]. A total of 20 mg of coarse drug was accurately weighed and mixed with 10 mL of 2% *w*/*v* poloxamer 188 by employing vortex mixing for adequate disaggregation of particles, followed by dispersion in 500 mL of water. The agitation of the Hydro^®^ cell was set at 2000 rpm for 3 min, after which the sample was further sonicated for 30 s. The samples were measured six times, and the results were expressed in terms of the De Brouckere and Sauter mean diameters ([D4,3] and [D3,2], respectively) and D10, D50 and D90.

### 2.5. Differential Scanning Calorimetry Analysis

Differential scanning calorimetry (DSC) studies of pure DFS, pure PVA (stabilizer), a physical mixture of DFS and PVA, and DFS-NS were carried out using a DSC Q100 (TA Instruments, Surrey, UK). Weighed samples of 3.0–5.0 mg were sealed in aluminum pans (nonhermetic). A flow rate of 50 mL per minute was set, and the heating rate was kept at 10.0 °C/min in nitrogen. To calibrate the DSC, the melting temperature of indium was set at 156.6 °C.

### 2.6. X-ray Diffraction Measurements

The study was carried out using a benchtop X-ray diffractometer (Miniflex™, Rigaku Corporation, Kent, UK). Radiation was from Ni-filtered Cu Kβ, with a wavelength of 1.39 Å at a voltage of 30 kV, a current of 15 mA, and at room temperature. DFS, PVA, a physical mixture of DFS and PVA and DFS-NS were packed into the rotating sample holder. The obtained data were typically collected by scanning a range of 0–60° with a scanning rate of 2°/min.

### 2.7. FTIR Measurements

Fourier transform infrared (FTIR) spectral analysis of DFS, PVA (10 kDa) and DFS-NS was conducted to study the drug–excipient interaction. The absorption spectra were recorded from 4000 to 400 cm^−1^ using an FTIR spectrometer (Accutrac FT/IR-4100™ Series, Jasco, Essex, UK).

### 2.8. Drug Content Analysis for DFS-NS

The drug content analysis for DFS-NS was performed in triplicate by dissolving accurately weighed (10 mg) freeze-dried NS into 1 mL of dimethyl sulfoxide (DMSO) and sonicating for 15 min. Then, 100 µL of the resulting solution was added to 900 µL of acetonitrile and centrifuged (12,000× *g*) for 10 min. Then, 100 µL supernatant was carefully collected and further diluted with 900 µL of phosphate buffer (PBS) containing 0.5% Tween 80 solution, followed by HPLC analysis. The % recovery of the drug was calculated using the following formula.

(1) $$\%\;\mathrm{Recovery}\;\mathrm{of}\;\mathrm{drug}\;=\frac{\mathrm{known}\;\mathrm{amount}\;\mathrm{loaded}}{\mathrm{amount}\;\mathrm{detected}\;\mathrm{via}\;\mathrm{analysis}}\times 100$$

### 2.9. In Vitro Release Studies for DFS NS

Release studies were performed using high retention cellulose dialysis tubing (2.3 mm, 0.9 inches). A total of 20 milligrams of accurately weighed drug and MNs were dispersed separately in 1 mL of PBS solution (in triplicate) and filled inside the dialysis membrane, secured with clips on both ends to contain the samples. These filled dialysis bags were then placed in 100 mL of PBS solution and incubated at 37 °C with mild shaking (40 rpm). A 500 µL sample of released media was taken at different time intervals of 2, 4, 6, 12, 24, 48, and 72 h, and 500 µL of the same media was replaced to maintain the volume. The samples were diluted by adding 500 µL of acetonitrile and centrifuged (2000× *g*) for 10 min to remove the polymer. Moreover, 100 µL of the supernatant was diluted with 900 µL of 0.5% of Tween 80 in PBS solution and injected into the HPLC for analysis.

### 2.10. Preparation of DFS-NS Loaded Dissolving Microneedles (DFS-NS DMN)

**Figure 2:**
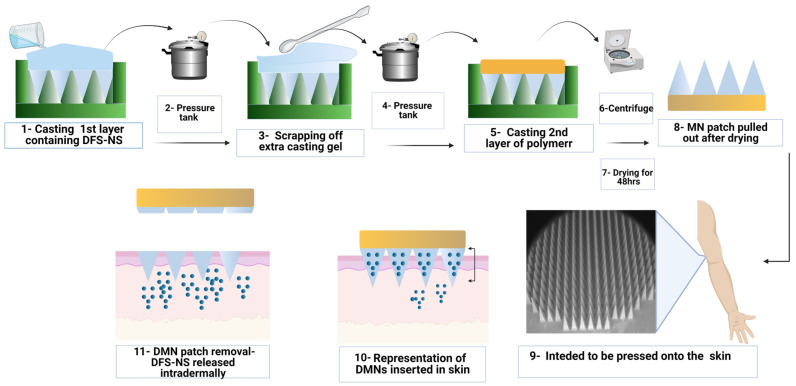
Schematic representation of the fabrication of DFS-NS DMNs and their application.

MN arrays were prepared using a silicone mold design, as presented in Figure 2, with microneedle heights of 700 μm, base widths of 300 μm and interspacing of 15 μm (a total of 600 arrays). These silicone molds were generously provided by LTS Lohmann (Germany). The polymers used for the preparation of the casting gel were PVA (9000–1000 MW) and PVP (K32/29). First, freeze-dried NS containing 300 mg of DFS was mixed with 1 ml of deionized water using a SpeedMixer™ (DAC 150.1 FVZ-K, Synergy Devices Ltd., UK) to form a homogeneous blend. This blend was then poured into 0.75 g of 40% PVP solution (K32/29). This mixture was homogenized again using SpeedMixer™ at 3500 rpm for 5 min to obtain a homogenous casting gel for MN fabrication. Here, 180 mg PVA is already present in the DFS-NS added. The DFS-NS casting gel was then poured onto the top surface of the MN molds and was subjected to a high-pressure tank at 60 psi for 3 min. The excess gel was removed by scraping lightly with a spatula, and the molds were again placed for 30 min in the pressure tank at the same pressure. After that, the molds were kept at room temperature for 24 h, and then 30% PVP (K90) solution was applied as a second layer (baseplate casting), followed by centrifugation (3500 rpm) for 8 min to remove any air bubbles. The MNs were removed from the molds after an additional 48 h of drying at room temperature and subjected to further studies.

### 2.11. Determination of Insertion Properties and Mechanical Strength of DFS-NS DMN Arrays

Parafilm M^®^, an elastic thermoplastic film made with a material resembling olefin (Bemis Company Inc., Soignies, Belgium), was used as a skin mimetic for the insertion of DFS-NS-DMNs. Initially, the height of the DMN arrays was recorded by stereomicroscopy prior to the application of compression force. For insertion studies, eight layers of Parafilm M^®^ sheet were placed onto the horizontal aluminum block under the movable probe and followed the same procedure as explained above. After the application of 32 N force, the DMNs were removed, and each layer of Parafilm M^®^ was observed under a microscope to count the number of holes created in each layer. The heights of DMNs after penetration into Parafilm M^®^ were noted using a Leica EZ4 D digital microscope to evaluate the reduction in height. The percentage insertion was calculated by the following formula:

(2) $$\mathrm{Percentage}\;\mathrm{insertion}=\frac{\mathrm{Number}\;\mathrm{of}\;\mathrm{holes}\;\mathrm{created}\;}{\mathrm{Number}\;\mathrm{of}\;\mathrm{microneedles}\;\mathrm{in}\;a\;\mathrm{patch}}\times 100$$

The mechanical properties of DFS-NS-DMNs and DFS-DMNs were studied using Texture Analyser (TA. XT-Plus, Stable Microsystem, Haslemere, UK) used in compression mode, as reported in earlier research [35,36]. The pre- and post-test speeds were 1 mm/s, while the trigger force was set at 0.049 N. A Leica EZ4 D digital microscope (Leica Microsystems, Wetzlar, Germany) was employed to study the morphological appearance of DFS-NS-DMNs before compression. Later, the DMN patches were fixed on the bottom of a movable probe using double-sided tape with needles facing down. The probe was run/declined against a horizontal, leveled block of aluminum at a rate of 0.5 mm s^−1^, and 32 N force was applied to the DMN patch for 30 s. After application of the desired force, DFS-NS-DMNs were examined for any size reduction using a Leica EZ4 D digital microscope, and the percentage reduction in height was calculated as follows.

(3) $$\%\;\mathrm{height}\;\mathrm{reduction}=\frac{\mathrm{mean}\;\mathrm{height}\;\mathrm{before}\;\mathrm{compression}-\mathrm{mean}\;\mathrm{height}\;\mathrm{after}\;\mathrm{compression}}{\mathrm{mean}\;\mathrm{height}\;\mathrm{before}\;\mathrm{compression}}\times 100$$

### 2.12. High-Performance Liquid Chromatography (HPLC) Analysis

Reversed phase HPLC (Agilent, 1260 Infinity II VWD, Germany) was used for analytical quantification of DFS using a C18 column (5 μm pore size, 4.6 × 100 mm) (Phenomenex, Macclesfield, UK). The flow rate was set at 1. ml/min, the oven temperature was set at 35 °C and analysis was performed with UV detection at 245 nm The mobile phase was composed of acetonitrile:phosphate buffer (pH 3.0), 50%:50%, and the injection volume was 10 µL. The standard calibration curve was plotted by making appropriate dilutions in the range of 0.096 to 100 µg/mL, and an R^2^ of 0.999 exhibited good linearity [37].

### 2.13. Drug Content Analysis for DFS-NS-DMNs

Drug content was analyzed by dissolving accurately weighed DFS-NS-DMN arrays as well as DFS-DMNs in 3 mL of water and sonicating for 15 min. Then, 2 mL DMSO was added, and the mixture was sonicated for 30 min for efficient extraction of the drug. Furthermore, 200 µL of this solution was mixed with 0.9 mL acetonitrile to allow precipitation of PVP polymer while the drug remained dissolved. This dispersion was centrifuged at 12,000× *g* for 10 min, and 100 µL of the supernatant was collected to further be diluted with 1.9 mL of mobile phase, which was then injected for HPLC analysis. Studies were performed in triplicate.

### 2.14. Digital Microscopy and SEM Imaging of DFS-NS-DMNs

The surface morphology and shape of DFS-NS-DMNs were examined using a Keyence VHX-700F Digital Microscope (Keyence, Osaka, Japan) and a TM3030 benchtop scanning electron microscope (SEM) (Hitachi, Krefeld, Germany). The latter was used in low vacuum mode at a voltage of 15 kV.

### 2.15. Insertion Studies in Excised Porcine Skin by Optical Coherence Tomography

It has been established earlier that the skin of neonatal pigs acts similarly to human skin [38]; hence, it was used to study the insertion of DFS-NS-DMNs. The skin was obtained from stillborn piglets and excised within 24 h of birth using a scalpel. The skin was then refrigerated at −20 °C for storage after being enclosed in aluminum foil until use. Prior to use, skin was defrosted and then thawed in phosphate-buffered saline (PBS) of pH 7.4, after which fine hair was removed carefully using a razor and washed thoroughly with PBS solution again. Absorbent tissue paper was used to dry the skin, and the skin was laid down flat on a weighing boat. Using the Texture Analyzer, DMN arrays were then pressed onto the porcine skin (force 32 N for 30 s). Optical coherence tomography (OCT) images were captured immediately upon insertion using an OCT Microscope (EX1301, Michelson Diagnostics Ltd., Kent, UK) to evaluate the successful insertion of MN arrays into the skin.

### 2.16. Dissolution Studies of DFS-NS-DMNs in Excised Porcine Skin

Dissolution of DMNs was determined by taking images by a Leica EZ4 D digital microscope at 10, 15, 30 and 60 min after insertion of the DMN patch into excised porcine skin following incubation at 37 °C. These images showed how much time DFS-NS-DMNs took to completely dissolve inside porcine skin. They also show the morphology of DMNs after each time point, depicting the gradual process of microneedle dissolution over time.

### 2.17. Ex Vivo Porcine Skin Deposition Study of DFS-NS-DMNs

Drug deposition for DFS-NS-DMNs was investigated using full thickness neonatal porcine skin, as described previously [34,39]. After thawing in phosphate-buffered saline (PBS) (pH 7.4), the skin was carefully shaved using a razor and washed with PBS before use. The skin surface was dried using tissue paper and placed dermis side down on paper sheets to provide support, and the underside of the skin was bathed in PBS (pH 7.4) at 37 °C for 30 min to equilibrate. After insertion of the MN patch, a cylindrical 12.0 g stainless steel weight was placed onto the top of the MN array patch to prevent MN expulsion and placed inside the oven at 37 ± 2 °C for 24 h. These pieces of skin along with the applied MNs were placed at 37 °C for 24 h. To prevent skin drying, another weighing boat was placed on the top, and 10 mL PBS (pH 7.4) solution was added to maintain skin hydration. Following applications, MNs remaining on the skin surface were carefully removed, and then the skin surface was thoroughly cleaned by applying 3 × 1 mL of PBS (pH 7.4) solution and gently wiped with wet paper tissue. The skin at the MN application site was then visualized using a Leica EZ4 W stereo microscope, and the MN-applied skin part was harvested using a scalpel. These harvested skins were cut into small pieces and placed into 2 mL Eppendorf tubes containing 0.5 mL water. The samples were bead milled using TissueLyser LT (QIAGEN^®^, Manchester, UK) for 15 min to solubilize the remaining MN shafts deposited in the skin. Subsequently, 1 mL of acetonitrile was added to each sample, and the mixture was homogenized for another 15 min again to solubilize the drug. The resulting mixture was then sonicated for 30 min and centrifuged at 48,000 rpm for 10 min to settle down the skin pieces. One hundred microliters of the supernatant was pipetted out into another 2 mL Eppendorf tube, and 900 µL of acetonitrile was added to it to precipitate out any polymers. After vortexing, the mixture was centrifuged again at 16,000 rpm for 10 min to settle down the precipitated polymer, and the supernatant was injected into the HPLC for analysis. Skin deposition studies were performed with DFS-NS-DMNs as well as pure drug-loaded DMN arrays. In addition, DFS-NS Transdermal patch (TP) and plain DFS-NS were investigated for drug deposition for comparison by following the same procedure as above.

## 3. Results and Discussion

DMN patches were fabricated with hydrophobic drug-loaded nanosystems to deliver the drug intradermally. DMN acts as a drug reservoir and is self-implanted subcutaneously to release drugs regionally and sustainably without producing systemic side effects. The DMN is applied on the skin surface and painlessly pierces the epidermis, creating microscopic aqueous pores through which drugs diffuse to the dermal microcirculation.

### 3.1. Characterization of DFS-NS

DFS-NS were prepared using the wet bead milling technique, and the pure drug was suspended in the medium with the help of a stabilizer. This method converted the pure drug into a nanosized range with a particle size in the 200 nm range. The particle size reduction increases the surface area and dissolution rate and, hence, enhances penetration of the drug through the skin [40]. A suitable stabilizer is necessary for producing a stable nanosuspension, as nanoparticles can be unstable due to higher Gibb’s energy, which interns because of their larger surface area [41].

PVA (9000–10,000 mol wt.) was selected as a stabilizer for the nanosuspension, as it is more compatible with polymeric microneedle arrays. Initially, 3% *w*/*w* PVA solutions (DFS-NS-1) were used as stabilizers, which were later reduced to 1% (DFS-NS-2) as the particle size and PDI were almost the same for both nanosuspensions. This can be attributed to the fact that a small change in the amount of surfactant seldom influences the particle size of the nanosuspension rather than the type of stabilizer, which is more important [42].

The bead size affected NSs formation to a great extent, as a rule of thumb exists that states that a 1000-fold particle size reduction is obtained in relation to the bead size. This seems to be true as a particle size in the range of 200 nm was obtained in the existing study with the 0.2 mm beads used. This indicates that bead size is directly proportional to particle size obtained after milling. This can be attributed to the fact that the ability of smaller beads to gnaw drug crystals is greater as they are more fast-moving. Nevertheless, extremely small beads are difficult to separate after milling and may also pose a risk of aggregation due to excess unutilized energy (not employed in size reduction) [43].

**Figure 3:**
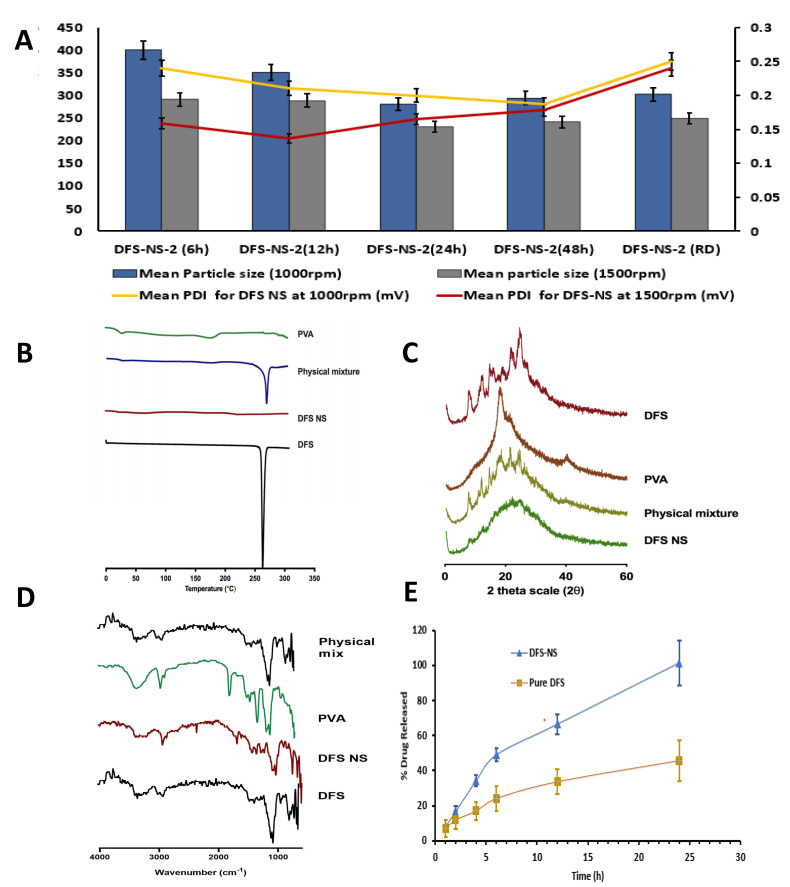
Particle size and PDI at 6, 12, 24, 48 h of milling and RD after freeze-drying at (**A**) 1000 rpm and 1500 rpm milling speed expressed as means + SDs, *n* = 3. (**B**) Differential scanning calorimetry thermogram of PVA, physical mixture of DFS and PVA, DFS-NS and DFS, (**C**) Powder X-ray diffraction of plain DFS, PVA, physical mixture of DFS and PVA and DF-NS, (**D**) Fourier transform infrared analysis of DFS, PVA, and DFS-NS. (**E**) In vitro release profile of DFS-NS and pure DFS by employing dialysis membrane and PBS as release media, expressed as the means ± SDs, *n* = 3.

Moreover, DFS-NS-2 was taken for DMN array fabrication, as higher drug loading is possible due to the lower concentration of PVA compared to the drug in the NS. With milling speeds of 1000 rpm and 1500 rpm, the mean particle sizes after 24 h of milling were 280.92 ± 18.72 nm and 230.92 ± 1.73 nm, respectively, as shown in Figure 3A. This clearly indicates that the milling speed has a huge impact on the particle size. The PDI was also improved from 0.20 ± 0.02 mV to 0.16 ± 0.02 mV after an increase in milling speed, indicating a more uniform size reduction at higher speeds.

The effect of milling time was also quite clear, as samples taken after 6 h of milling (at 1500 rpm speed) exhibited a particle size of 291.32 ± 4.54 nm with a PDI of 0.159 ± 0.020 mV, while at 24 h, it was further reduced to 230.92 ± 1.02 nm with a PDI of 0.125 ± 0.023 mV. Therefore, particle size was reduced as a function of milling time, as samples taken at 6 h, 12 h, and 24 h showed a consistent reduction in particle size, as shown in Figure 3B, with an almost constant PDI below 0.2. However, the particle size did not show any considerable reduction in samples taken at 48 h of milling, which shows that 24 h of milling is more suitable for efficient and maximum particle size reduction. Moreover, the particle size increased in most of the samples to 240.92 ± 4.03 nm after 48 h of milling because further exposure to high energy increases the kinetic energy of the crystalline material, resulting in aggregation rather than a reduction in size.

The zeta potentials for both DFS-NS-1 and DFS-NS-2 were −19.46 mV ± 1.56 and −20.51 mV ± 3.46, respectively, which shows that PVA formed a surface adsorption layer on the particles, preventing aggregation and therefore providing sufficient stabilization. After freeze-drying, the particle size and PDI were checked, and a slight increase in particle size (248.95 nm ± 10.81) was observed. The zeta potential value of redispersed (RD) DFS-NS-2 was −19.46 mV ± 5.61, which shows good stability. The particle size profile of DFS showed a wider particle size range than that of DFS-NS-2, which clearly indicates that the DFS-NS particle size is more uniformly distributed. DFS had a particle size of D [2,3,4,5,6,7,8,9,10,11,12,13,14,15,16,17,18,19,20,21,22,23,24,25,26,27,28,29,30,31,32,33,34,35,36] 2.46 µm, D [9] 6.71 µm, Dv (10) 1.12 μm, Dv (50) 3.90 μm, and Dv (90) 7.98 µm. DFS-NS-2 was selected for further formulation into DMNs, which will be referred to as DFS-NS-DMN in the upcoming text.

### 3.2. Characterization Using Differential Scanning Calorimetry, X-ray Diffraction and FTIR

The physical state of DFS before and after being manufactured into NS was determined by DSC. As shown in Figure 3B, the DSC thermogram of DFS exhibited a characteristic sharp endothermic peak at 260 °C, which corresponds to the melting point of DFS in the crystalline state. This characteristic peak was observed in the physical mixture but was absent in the lyophilized DF-NS, which shows its amorphous nature.

To verify the obtained DSC results and to reconfirm the crystalline state of the lyophilized DFS-NS, XRD analysis was carried out, and the peaks of DFS, PVA, and the physical mixture of DFS and DFS NS are presented in Figure 3C. The diffractogram of pure DFS displayed several sharp peaks at the diffraction angles (2θ) of 10.47°, 14.61°, 23.97° and 26.67°, indicating that DFS is present in a crystalline form. In contrast, there were no distinguished peaks in the XRD diffractograms of the NS form, indicating that the NS formulations present an amorphous structure. Overall, when taking both the DSC and XRD results into account, it can be concluded that the crystalline structure of DFS was largely amorphized following bead milling.

The FTIR spectra of DFS showed the presence of peaks at ~1750 cm^−1^ indicating the presence of the carboxylic acid group, along with the presence of a peak at ~1100 cm^−1^ confirming the presence of phenyl structure. The peak at ~3350 cm^−1^ prominently indicated a phenol hydroxyl group. Similar peaks were observed in DFS NS samples confirming the presence of DFS. However, DFS NS also showed the characteristic peaks of PVA ~2900 cm^−1^ and ~2850 cm^−1^ indicating the asymmetric and symmetric C-H stretching vibrations which could also be observed in the PVA sample. The FTIR spectra of DFS NS presented the prominent peaks of Both DFS and PVA; however, no peak shifting and generation of the new peak was observed, suggesting limited chemical and physical interaction between both chemicals. The FTIR spectra of the physical mix showed the presence of characteristic peaks of DFS and PVA; however, no major change in the peak was observed to indicate any chemical interaction between DFS and PVA.

SEM images were obtained for DFS, DFS-NS and DFS NS-loaded MN tips by SEM with a Quanta FEG 250 (FEI, Hillsboro, OR, USA). SEM images showed that DFS-NS-DM arrays were formed well structurally. The resulting needles measured 700 μm in height and displayed sharp tips.

### 3.3. Drug Content and In Vitro Release Study

The percentage recovery of the drug in DFS-NS was found to be 85% ± 6.5%, which suggested that some drug was lost while separating the nanosuspension from the milling beads. A dialysis membrane is widely used for the release study of various drugs, and a skin condition is critical for the true assessment of drug release, independent of saturation effects and dissolution media volume. The skin condition was maintained at three times the volume of dissolution media (PBS) compared to the solubility of DFS in PBS. The drug release kinetics of DFS-NS were conducted to assess whether nanosizing led to an increase in the dissolution rate. The dissolution profile of the pure drug dispersed in PVA (prepared using the same method as adopted for DFS-NS) exhibited a lower dissolution rate, as only 40.6% of the drug was solubilized/released into the media after 24 h, whereas the DFS-NS showed 99.89% release until 24 h, as shown in Figure 3E. This can be attributed to the reduction in particle size, which increases the specific surface, which, according to the Noyes–Whitney equation, leads to an increase in the dissolution rate [25]. Many other reports in the literature have shown similar results for poorly soluble drugs [44]. Moreover, the wettability and saturation solubility of the drug in the nanosuspension form are also increased [20,45,46].

### 3.4. Characterization of DFS-NS-DMN Arrays

An aqueous hydrogel of PVA and PVP was used in combination to prepare the DMN casting gel. PVA and PVP have been extensively employed for DMNs owing to their hydrophilicity, biocompatibility, and strength for durable fabrication. Additionally, densely packed DMNs are formed due to their adhesive property. PVA alone caused DMNs to bend because they were too soft, while too much PVP triggered the brittleness of needles. The correct combination of the two was found in 30% *w*/*w* PVA and 20% *w*/*w* PVP.

The aqueous blend was prepared by adding different concentrations of lyophilized DFS-NS to attain the highest achievable drug loading for the system while maintaining the strength of the DMN arrays. For the second layer, only PVP without drug was used, as previous studies have shown that drugs in the baseplate, as well as needles, result in poor mechanical strength. Moreover, it helps in the efficient use of the drug, as drugs in the baseplate seldom deposit inside the skin [47,48].

**Figure 4:**
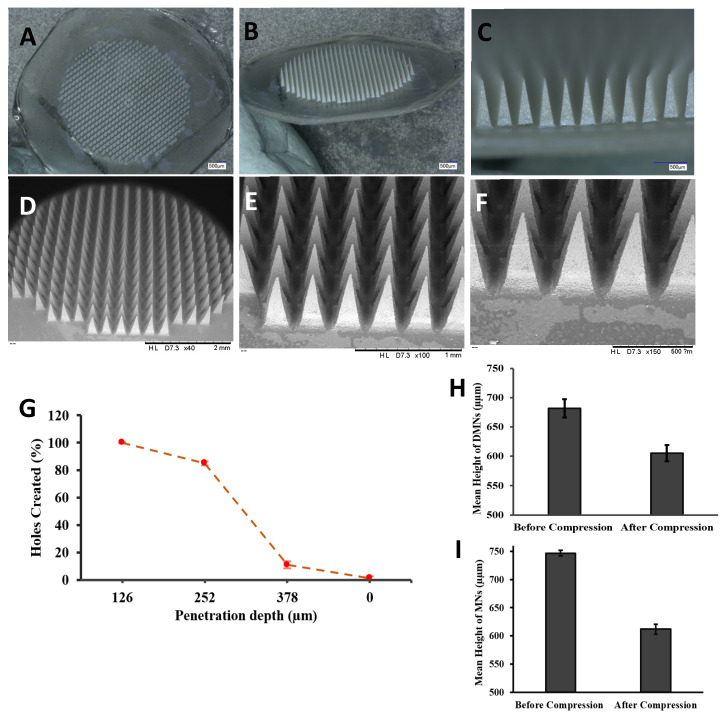
(**A**–**C**) Digital images of DFS-NS-DMNs at different magnifications. (**D**–**F**) SEM images of DFS-NS-DMNs. (**G**) The percentage of holes created in Parafilm M^®^ layers and the corresponding approximate insertion depth. Mechanical strength determination of DMNs by a texture analyzer by applying a force of 32 N for 30 s (mean ± SD, *n* = 6). (**H**) Mean height reduction of DFS-NS-DMNs, (**I**) Mean height reduction of DFS-DMNs.

The formation of homogenous MN arrays with sharp tips was confirmed by viewing them under a microscope with a length of 700 µm. The morphology was further investigated using a digital microscopy and SEM imaging, as shown in Figure 4A–F, displaying uniform needle formation throughout the MN patch. The mean drug loading in the MN array was 1459.20 ± 15.44 µg drug in an entire MN array of 700 needles, which means 2.08 µg in each needle. The particle size and PDI were measured by dispersing in purified water each time before (248.95 nm ± 10.81) and after (291.13 nm ± 12.72) loading the DFS-NS into the DMN arrays.

### 3.5. Determination of Insertion Properties and Mechanical Strength of DFS-NS DMN

The insertion properties of the DMNs were also adequate, as the needles penetrated three layers of Parafilm M^®^ with 100% penetration in the first layer, 85% in the second and 15% in the third layer, as revealed in Figure 4G. It has already been proven that penetration up to 330 μm is insufficient for effective deposition of drugs across the skin. The thickness of each layer of Parafilm M^®^ was approximately 126 μm. There was no height reduction, and the needle length that was inserted was approximately 378 µm, which is equal to 56% of the total height of the MNs. Meanwhile, DFS-DMN penetrated up to two layers.

**Figure 5:**
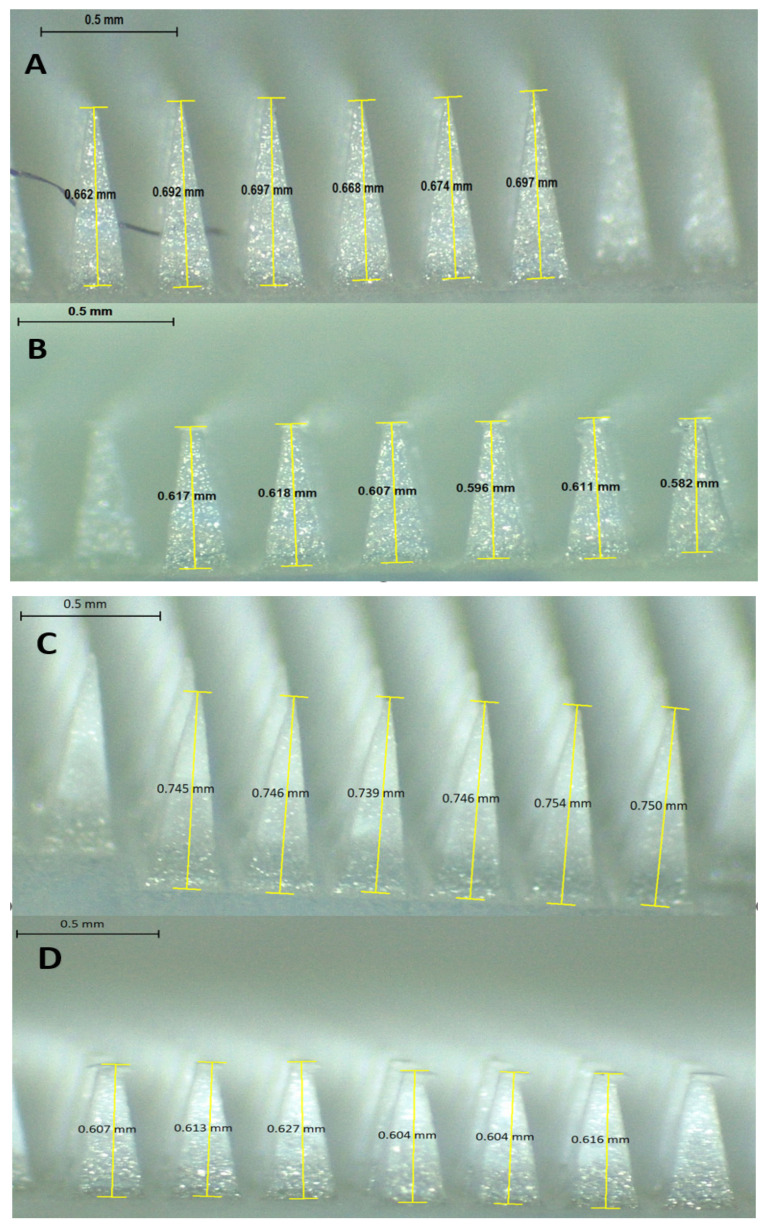
Mechanical strength determination of DMNs by a texture analyzer by applying a force of 32 N for 30 s (mean ± SD, *n* = 6). (**A**) Heights of DFS-NS-DMNs before compression; (**B**) Heights of DFS-NS-DMNs after compression; (**C**) Heights of DFS-DMNs before compression; (**D**) Heights of DFS-DMNs after compression.

The reduction in size of the DFS-NS-DMNs was calculated to be 11.5%, as shown in Figure 4H and Figure 5A,B. This value depicts excellent strength, as the needles were able to bear the 32 N force without exhibiting a loss in height of more than 11.5%. It has been established previously that 32 N equates to the mean force applied by human subjects to insert microneedles into their skin. The DFS-DMNs exhibited an 18% (Figure 4I and Figure 5C,D) reduction in the height of needles after compression, which shows inadequate strength to be inserted into human skin. Therefore, DFS-DMNs will likely fail to deliver drugs intradermally because of the lack of sufficient mechanical strength required for penetration into the skin.

### 3.6. Scanning Electron Microscopy

**Figure 6:**
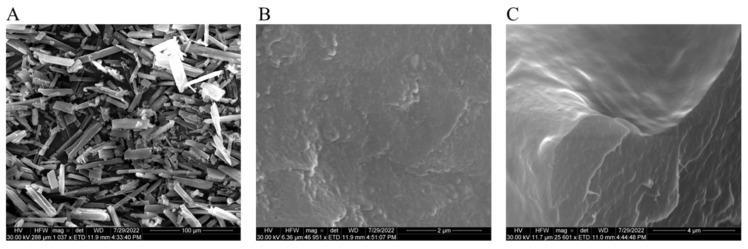
Scanning electron micrographs of (**A**) DFS crystals, (**B**) freeze-dried DFS-NS before DMN loading, and (**C**) magnified image of DFS-NS embedded into the DMN tips.

SEM images showed the acicular crystalline structure of pure DFS (Figure 6A) with quite large particle size (20 µm to 50 µm), while freeze-dried DFS-NS in Figure 6B depicted the spherical particle size with sizes ranging from approximately 200 nm. SEM image (Figure 6C) of broken single MN tip with exposed outer surface (upper half image) as well as internal cross-section (lower half image) showed a smooth surface and uniform distribution of DFS-NS in the PVA/PVP DMN matrix, respectively, without any evident particle aggregation.

### 3.7. Dissolution of DFS-NS-DMN Arrays

**Figure 7:**
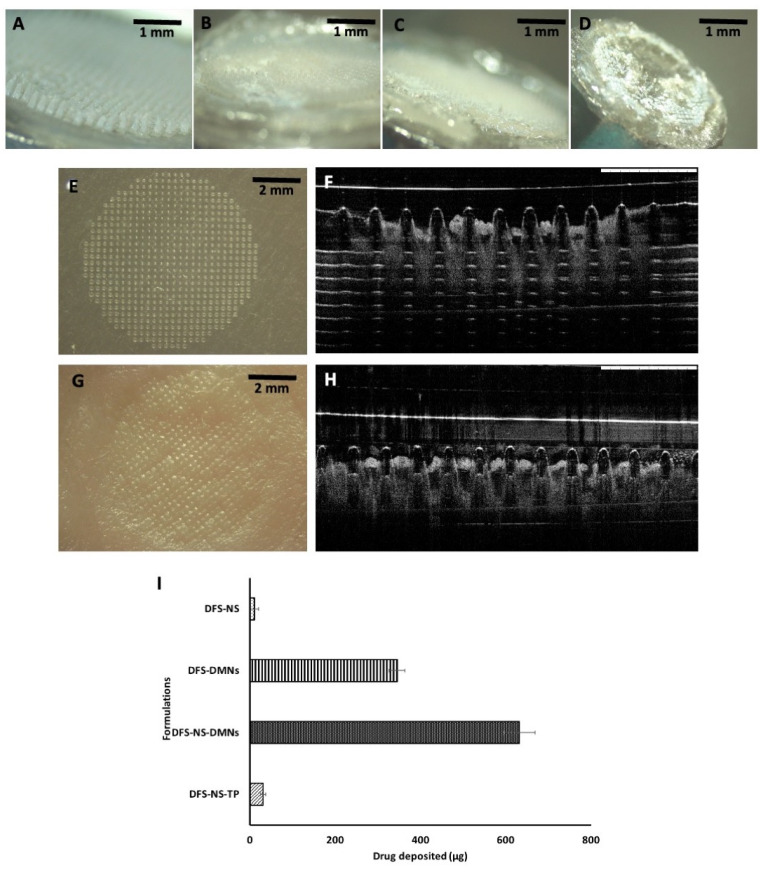
Dissolution study of DFS-NS-DMNs after insertion into excised porcine skin. Images taken after (**A**) 10 min of insertion, (**B**) 15 min of insertion, (**C**) 30 min of insertion, and (**D**) 60 min of insertion. Digital and OCT images of DFS-NS-DMNs; (**E**,**F**) DFS-NS-DMNS inserted in parafilm layers, (**G**,**H**) DFS-NS-DMNs inserted into excised porcine skin. (**I**) DFS deposited in excised porcine skin following the insertion of DFS-NS, DFS-DMNs, DFS-NS-DMNs, and DFS-NS-TP (without needles). Data are expressed as the mean ± SD, *n* = 3.

Dissolution studies of the DFS-NS-DMN array were performed using porcine skin. The DMNs were applied for 60 min, and the dissolution time of DMNs was observed at different time intervals of 10, 15, 30 and 60 min. DMNs were completely dissolved within 30 min, as shown in Figure 7A–D, revealing that DMNs were fabricated properly. The rapid dissolution profile of these MAP formulations in intradermal fluid may be attributed to the hydrophilic nature of the needles with the easily dispersible nanosuspension form of DFS.

### 3.8. Determination of Skin Penetration

OCT images revealed how DFS-NS-MNs were inserted into the parafilm layers as well as excised porcine skin [49,50]. These arrays were capable of penetrating to a great extent through the layers of parafilm (Figure 7E,F) and porcine skin (Figure 7G,H). A significant length of microneedles can be seen inside the parafilm layers, and needles were inserted up to the 4th layer (378 µm), which reiterates what was observed previously in penetration studies. The holes created on porcine skin can be seen in Figure 7G. The skin is flexible in nature, so the pores seem to close up after a while, leaving a print of the inserted DMNs behind.

### 3.9. Deposition Study on Excised Porcine Skin

The deposition study indicates the amount of drug that is deposited inside the skin from the DFS-NS-DMNs. The mean drug content deposited inside the skin was 632.47 ± 16.5 µg, comparing approximately 60% of the total drug loaded in DMNs. Drug deposition from DFS-DMNs into skin was calculated as 345 ± 12.5 µg. This clearly indicates that a greater amount of drug was deposited into porcine skin from DFS-NS-DMNs. This can be due to a greater dissolution rate of DFS-NS into the interstitial fluid of porcine skin compared with pure DFS, as demonstrated earlier in release studies. This might also be due to higher drug loading into DMNs, since nanosuspensions of hydrophobic drug allow a more homogeneous loading along the entire length of polymeric microneedles compared to the pure hydrophobic drug [9]. Pure DFS has a particle size of D [9] 2.46 µm, D [9], 6.71 µm Dv (10), 1.12 μm, Dv (50) 3.90 μm and Dv (90) 7.98 µm, as measured by DLS, and the results are expressed in terms of the De Brouckere and Sauter mean diameters, which are much larger than the DFS-NS particle size (248 nm).

The amount of drug deposited from DFS-NS-TP (without needles) was 30.56 ± 3.56 µg, and the amount of drug deposited by the application of DFS-NS alone was only 10 ± 4.32 µg. These values indicate that DMNs can deliver a significantly larger amount of drug as they can penetrate the stratum corneum, unlike DFS-NS-TP and DFS-NS, as shown in Figure 7I.

Therefore, the DMN facilitates higher DFS delivery across the skin. Additionally, the reduced particle size of DFS allows the homogeneous distribution of DFS into the DMN lower end of the tips, which also greatly influences drug deposition in the skin. This NS form of DFS also allows for increased surface area, greater dissolution rates and subsequently better absorption intradermally.

Nevertheless, the drug deposition study conducted in this research from full-thickness porcine skin did not depict the level of drug in each layer of the skin (SC, epidermis and dermis); therefore, it is difficult to predict the DFS gradient and availability of DFS for immediate systemic release or sustained effect of the DFS. However, based on previous work from our research group with similar type of hydrophobic drugs (cabotegravir, rilpivirine), DFS NS deposited intradermally with dissolution as well as skin rate-limiting factor for the sustained release [51,52,53,54] could allow the reduction of the frequency of DFS dose administration. However, to prove the sustained delivery effect, further in vivo research is needed.

In the current work, we successfully formulated dissolving MAPs loaded with an optimized NS of DFS. These formulations have been shown to display acceptable mechanical properties enabling effective skin penetration, as evidenced from the Parafilm^®^ M and ex vivo skin insertion study. To develop an MN-based formulation for the management of iron toxicity, we prefer the dissolving MN approach relative to other types of MNs [49,55]. With a dissolving MAP approach, we can easily administer DFS in a single-step application process with a short wear time. This single MN application with delivered dose could be high enough for localized site-specific application. However, single MN would not be adequate for use in humans to deliver the enough systemic dose to get the therapeutic response; therefore, multiple MN within one larger patch need to be formulated and investigated in vivo to prove the usefulness of this delivery route in iron toxicity management [56]. 

## 4. Conclusions

An iron chelator DFS was incorporated for the first time in DMNs successfully in the form of a nanosuspension, exhibiting appropriate mechanical strength for effective skin insertion. Deposition studies revealed that DFS can be proficiently deposited into porcine skin for local and possible systemic delivery without the use of hypodermic needles and intervention of healthcare professionals, as well as evading side effects of the oral route. Moreover, DFS-NS showed a greater dissolution rate than pure DFS for probable subsequent uptake by the rich dermal microcirculation. Thus, the results support the claim that DFS-NS-DMNs can prove to be significant alternatives to conventional routes of delivery. This proof-of-concept study provides the basis for further investigation through in vivo studies to explore the therapeutic efficacy and expansion of this work by forming larger DMN patches for loading higher drug doses.

## Data Availability

Not applicable.
